# Tsukushi Modulates Xnr2, FGF and BMP Signaling: Regulation of *Xenopus* Germ Layer Formation

**DOI:** 10.1371/journal.pone.0001004

**Published:** 2007-10-10

**Authors:** Samantha A. Morris, Alexandra D. Almeida, Hideaki Tanaka, Kunimasa Ohta, Shin-ichi Ohnuma

**Affiliations:** 1 Department of Oncology, The Hutchison/Medical Research Council Research Centre, University of Cambridge, Cambridge, United Kingdom; 2 Department of Developmental Neurobiology, Graduate School of Medical Sciences, Kumamoto University, Kumamoto, Japan; Max Planck Institute of Molecular Cell Biology and Genetics, Germany

## Abstract

**Background:**

Cell-cell communication is essential in tissue patterning. In early amphibian development, mesoderm is formed in the blastula-stage embryo through inductive interactions in which vegetal cells act on overlying equatorial cells. Members of the TGF-β family such as activin B, Vg1, derrière and *Xenopus* nodal-related proteins (Xnrs) are candidate mesoderm inducing factors, with further activity to induce endoderm of the vegetal region. TGF-β-like ligands, including BMP, are also responsible for patterning of germ layers. In addition, FGF signaling is essential for mesoderm formation whereas FGF signal inhibition has been implicated in endoderm induction. Clearly, several signaling pathways are coordinated to produce an appropriate developmental output; although intracellular crosstalk is known to integrate multiple pathways, relatively little is known about extracellular coordination.

**Methodology/Principal Findings:**

Here, we show that *Xenopus Tsukushi (X-TSK)*, a member of the secreted small leucine rich repeat proteoglycan (SLRP) family, is expressed in ectoderm, endoderm, and the organizer during early development. We have previously reported that X-TSK binds to and inhibits BMP signaling in cooperation with chordin. We now demonstrate two novel interactions: X-TSK binds to and inhibits signaling by FGF8b, in addition to binding to and enhancement of Xnr2 signaling. This signal integration by X-TSK at the extracellular level has an important role in germ layer formation and patterning. Vegetally localized X-TSK potentiates endoderm formation through coordination of BMP, FGF and Xnr2 signaling. In contrast, X-TSK inhibition of FGF-MAPK signaling blocks ventrolateral mesoderm formation, while BMP inhibition enhances organizer formation. These actions of X-TSK are reliant upon its expression in endoderm and dorsal mesoderm, with relative exclusion from ventrolateral mesoderm, in a pattern shaped by FGF signals.

**Conclusions/Significance:**

Based on our observations, we propose a novel mechanism by which X-TSK refines the field of positional information by integration of multiple pathways in the extracellular space.

## Introduction

During blastula stages of amphibian development, the embryo is organized into three distinct germ layers: ectoderm, mesoderm and endoderm, precursors of skin, connective tissue and gut respectively. Mesoderm is formed through inductive interactions by which cells of the vegetal region act upon overlying equatorial cells (Reviewed in [1]), an event relying upon pre-localized maternal determinants. Moreover, these overlying cells receive differential signals from specific vegetal areas, resulting in initial ventral-dorsal patterning of the embryo [2]. The vegetal cells themselves are destined to become endoderm, in a process that has not been as intensively studied, in comparison to mesoderm formation. Formation of the endoderm begins during early blastulation, where gene expression specific to this germ layer is reinforced by late blastula stages [3] and interestingly, the process of endoderm formation involves signals shared with the events of mesoderm induction and patterning.

Cell-cell communication is essential in germ layer formation and patterning. Studies on mesoderm formation have identified several mesoderm-inducing factors; Activin B [4], Vg1 [5], Derrière [6] and *Xenopus* nodal-related proteins (Xnrs), [7], [8] members of the activin-like branch of the Transforming Growth Factor-β (TGF-β) family of signaling molecules. These proteins act as morphogens and demonstrate activity to induce dorsal mesoderm formation at higher concentrations [9], [10]. A second, larger branch of the TGF-β family comprising of Bone Morphogenetic Proteins (BMPs) function to ventralize mesoderm, highlighting the importance of TGF-β signaling in both mesoderm formation and its subsequent pattering (Reviewed in [11]).

Interestingly, activin-like TGF-β signals are also essential components in the mechanism of endoderm induction. Vegetally localized T-box transcription factor (VegT) is essential for initiation of endoderm formation by activating expression of TGF-β-related Xnr family members and Derrière, in addition to endoderm-specific transcription factor Sox17 [12]–[17]. This occurs at the early blastula stage and functions to achieve a high level of activin-like TGF-β signaling within presumptive endoderm [13], [18]. *Sox17* expression activated early by VegT later relies upon Xnr signals for maintenance of its expression [19], and by late blastula stages, expression of endoderm specific transcription factors of the Mix and GATA families is induced (reviewed in [3]). Upon gastrulation, mesodermal and endodermal cell populations become distinct [20], yet is not known how a clear border is made between these segregated cell populations when common signaling pathways are employed for their formation and maintenance.

**Figure 1:**
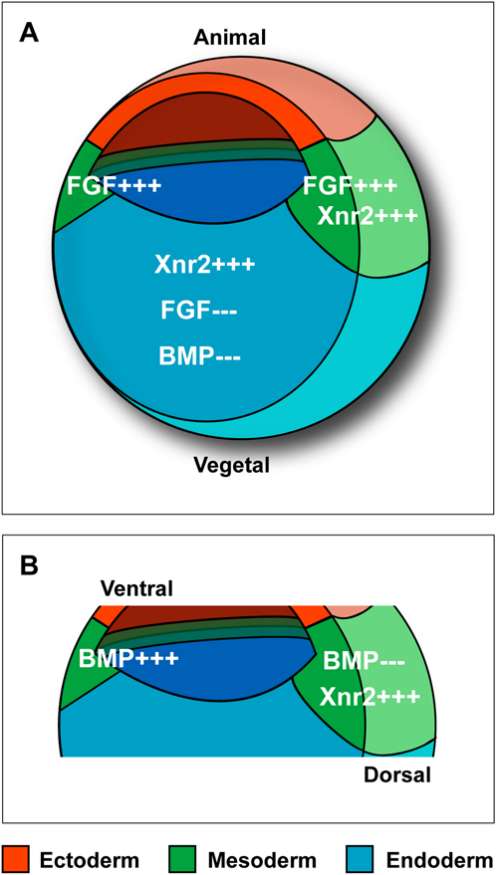
Signaling involved in *Xenopus* germ layer formation. (A) Selected signaling pathways involved in *Xenopus* mesoderm and endoderm formation. Activation of pathways indicated by ‘+++’, inhibition of pathways indicated by ‘−−−‘. Ectoderm = red, mesoderm = green, endoderm = blue. FGF signal activity is required for mesoderm formation in addition to activity of activin-like signaling (represented here by Xnr2). FGF and BMP signal inhibition with Xnr2 signal activation is involved in endoderm induction mechanisms. (B) Selected signaling pathways involved in *Xenopus* mesoderm patterning. Active BMP signaling produces mesoderm with ventral character, whereas inhibition of BMP signaling produces mesoderm of dorsal character. Also, Xnr2 expressed in the dorsal region has activity to induce dorsal mesoderm.

It is clear that morphogen gradients form part of the mechanism responsible for differential gene expression in the *Xenopus* embryo [21]. Additional signaling pathways may be accessory to differentiation between germ layers. For example, intact FGF signaling is essential for mesoderm induction [22], [23], which is integrated with activin-like signaling [24], [25]. In contrast to this, FGF signal inhibition has been implicated in endoderm formation [26]. Similarly, active BMP signaling is involved in ventral mesoderm formation, whereas BMP inhibition is linked to endoderm formation [26], [27]. These germ-layer specific signal requirements are clarified in Figure 1, illustrating the point that in order to achieve precise germ layer specification, multiple signaling pathways initiated by extracellular molecules must be coordinated to produce an appropriate output. Intracellular cross-talk has been intensively studied in the context of signal integration; for example, activation of MAP kinase, downstream of FGF signaling, inhibits BMP through phosphorylation of the Smad1 linker domain [28]. There is now increasing evidence that individual extracellular regulators interact with multiple morphogens, such as Cerberus (Wnt, Xnrs, BMP), Coco (Wnt, Xnrs, BMP) and Follistatin (Activin, BMP) [29]–[32], suggesting that extracellular regulators play an important role in integration of multiple pathways.

We have previously reported that a short-range secreted protein, Tsukushi (TSK), interacts with and modulates activities of TGF-β family members, BMP and chick Vg1, in addition to the Notch ligand, Delta [33]–[35]. We aimed to study multiple signal regulation in early development, coordinated at the extracellular level by an individual type of molecule. In this study, we show that *Xenopus* Tsukushi (X-TSK) plays an important role in multiple signal integration, through binding and modulation of BMP, FGF and Xnr2. Zygotic *X-TSK* expression is activated in endoderm and the dorsal blastopore lip, with relative exclusion from ventrolateral marginal zone. Functional analysis shows that X-TSK potentiates endoderm formation whilst inhibiting ventrolateral mesoderm formation, through activation of Xnr2 signaling combined with inhibition of FGF and BMP signaling. Indeed, through FGF-dependent localized expression of *X-TSK* and coordinated regulation of three distinct signaling pathways, TSK contributes to germ layer formation and patterning in *Xenopus* development.

## Results

### *X-TSK* is expressed in ectoderm, dorsal mesoderm and endoderm

We previously demonstrated that TSK functions in primitive streak and Hensen's node formation through modulation of BMP and Vg1 in chick [33]. In *Xenopus* embryos, Chick TSK (C-TSK) induces dorsal mesoderm formation, site of the organizer, most likely through BMP inhibition. Although we have previously shown a role for TSK in neural crest formation in *Xenopus* through BMP and Notch signal modulation [34], we have yet to detail any possible multiple signal interactions during earlier stages of amphibian development.

**Figure 2:**
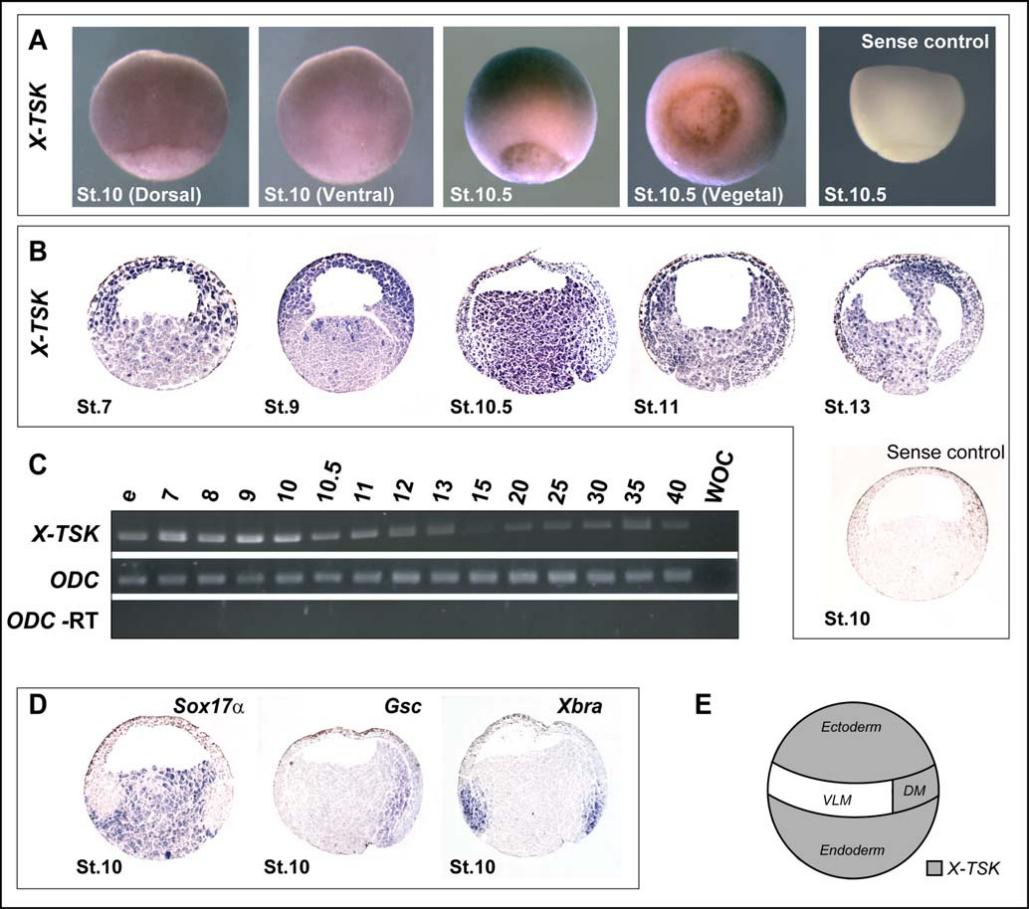
*X-TSK* expression in *Xenopus.* (A) Whole mount in situ hybridization of *X-TSK* in *Xenopus* gastrula stage embryos, including sense control. Purple staining indicates *X-TSK* expression. Orientations and stages as indicated. *X-TSK* is expressed in dorsal marginal zone (DMZ) and ectoderm from stage 10, and endoderm from stage 10.5. (B) In situ hybridization of *X-TSK* in sectioned *Xenopus* embryos, including sense control. Orientation: animal top, vegetal bottom, dorsal right, stages as indicated. *X-TSK* is expressed maternally (stage 7) in the animal region, with light staining in the vegetal region. From stage 10.5, *X-TSK* expression is detected throughout the endoderm. (C) Expression levels of *X-TSK* (upper panel) measured by RT-PCR from egg to stage 41, including *ODC* expression (middle panel) and -RT control (lower panel). WOC = Water Only Control. *X-TSK* is expressed at highest levels during germ layer formation and gastrulation. (D) Comparative expression of *Sox17α* (marking endoderm), *Gsc* (dorsal mesoderm), and *Xbra* (pan-mesoderm) in sectioned stage 10 embryos. (E) Schematic of *X-TSK* expression (grey) in ectoderm, dorsal mesoderm and endoderm.

In order to learn more about TSK function in early *Xenopus* development, *X-TSK* mRNA expression was examined spatially and temporally by in situ hybridization and RT-PCR. Whole mount in situ hybridization showed that *X-TSK* is localized to the animal hemisphere during late blastula and gastrula stages, as identified by purple staining (Figure 2A). At stage 10, *X-TSK* expression is also detected around the dorsal blastopore lip, relative to lower expression in the ventrolateral marginal zone. Furthermore, punctate staining within the fully formed blastopore is evident from stage 10.5, suggesting localization of *X-TSK* to the endoderm. Staining is not observed with sense *X-TSK* probe, confirming that *X-TSK* staining is specific.

As probe cannot penetrate into deep endodermal tissue by whole mount in situ hybridization, due to large size of the embryo and high yolk content [36], embryos were first sectioned, followed by in situ hybridization to examine TSK expression within the vegetal region. Figure 2B demonstrates that *X-TSK* mRNA is expressed in the pre mid-blastula transition (MBT, stage 7) embryo. This maternal expression is localized to the animal hemisphere, in addition to a small amount of vegetal expression in the blastocoel floor. At stage 10.5, zygotic expression of *X-TSK* is detected throughout the vegetal region and the dorsal blastopore lip, overlapping with expression of the endoderm-specific marker *Sox17α* [37] and with the organizer marker *Goosecoid (Gsc),* in the dorsal marginal zone [38] (Figure 2D). In contrast to this, expression is diminished in the area where the pan-mesoderm marker, *brachyury* (*Xbra)*, is expressed [39] (Figure 2D). Later in gastrulation (stage 13), *X-TSK* is strongly expressed in the endodermal derived region and precaudal plate under the neuroectoderm (Figure 2B). In order to demonstrate the specificity of staining for *X-TSK* expression, in situ hybridization with sense probe was again performed, resulting in minimal staining as shown in Figure 2B.

Temporally, *X-TSK* expression levels peak during germ layer formation and early gastrulation, as demonstrated by semi-quantitative RT-PCR in whole embryos (Figure 2C). These data showing the specific expression of *X-TSK* in ectoderm, endoderm and dorsal mesoderm during early stages of *Xenopus* development suggest a function for X-TSK in germ layer formation and patterning.

### Loss-of-function: X-TSK is a component of endoderm formation and mesoderm patterning

In order to determine the importance of X-TSK in *Xenopus* germ layer formation and patterning we depleted X-TSK with antisense morpholino oligonucleotide (XMO) targeted to prospective endoderm or mesoderm. We have previously shown that this MO specifically depletes X-TSK protein levels [34], [40], where morphants were analyzed from stage 15, and exhibited a reduction in neural tissue formation, although earlier stages have not previously been analyzed.

**Figure 3:**
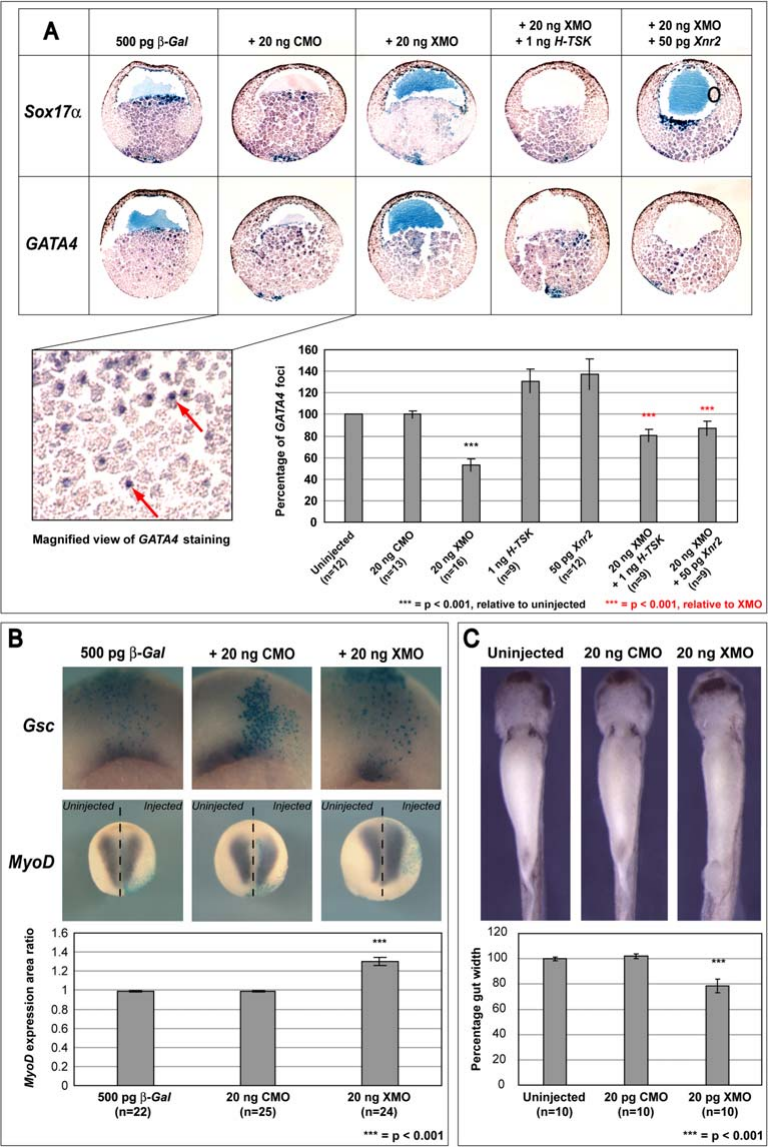
Loss of X-TSK function. (A) In situ hybridization of endoderm markers, *Sox17α* (upper row), and *GATA4* (lower row) in sectioned early gastrula (stage 10) embryos, purple staining indicates expression. Orientation: animal top, vegetal bottom. All embryos injected with 500 pg *β-Galactosidase (β-Gal)* to identify targeted area (blue staining), with 20 ng control morpholino (CMO) or 20 ng X-TSK morpholino (XMO). Endoderm marker staining is reduced in XMO injected embryos, as indicated by general loss of purple staining (*Sox17α*) and loss of punctate staining (*GATA4*), detailed in the zoomed panel. Rescues were performed with 1 ng *H-TSK*, or 50 pg *Xnr2*, restoring endoderm marker expression. Detailed analysis of *GATA4* staining in sectioned embryos. Numbers of *GATA4* foci were counted, as represented graphically, relative to uninjected control. XMO injection reduces *GATA4* foci by 50% (p = <0.001), partially rescued by 1 ng *H-TSK* and 50 pg *Xnr2* to over 80% relative to control (p = <0.001). (B) Whole mount in situ hybridization of dorsal mesoderm marker, *Gsc* in stage 10.5 embryos (dorsal orientation) and *MyoD* in stage 16 (anterior top, posterior bottom) in embryos injected with 500 pg *β-Gal,* with 20 ng CMO or 20 ng XMO. *Gsc* expression is reduced in XMO injected embryos, whereas *MyoD* expression is expanded by 30% (relative to control, p = <0.001) on the injected side, as identified by blue *β-Gal* staining. (C) Gut morphology in stage 40 embryos injected with 20 ng CMO or 20 ng XMO. Gut width is reduced by 21% in XMO injected embryos, relative to uninjected embryos (p = <0.001).

As we found *X-TSK* to be expressed throughout the vegetal region from gastrula stages, with a small amount of expression in the pre-gastrula embryo, we targeted XMO to prospective endoderm, followed by in situ hybridization analysis of endoderm markers *Sox17α* and *GATA4* [37], [41]. In addition to performing this analysis in the whole embryo, we again subjected sectioned embryos to in situ hybridization in order to access the deep endodermal tissue. XMO was co-injected with *β-Galactosidase* RNA to facilitate identification of the targeted area, as observed from the region of light blue staining. As part of these loss-of-function approaches, we also injected control MO (CMO) to rule out non-specific effects of MO on development. *Sox17α* and *GATA4* are expressed throughout the vegetal region of gastrula-stage embryos, with punctate staining of *GATA4* observed as highlighted in the zoomed panel (Figure 3A). Expression of both *Sox17α* and *GATA4* are visibly diminished upon X-TSK depletion with 20 ng XMO in 41% and 48% of embryos respectively (Figure 3A and Table 1). Punctate *GATA4* staining is reduced in the presence of XMO, which has enabled quantification of loss-of-function by counting what we have termed as *GATA4* positive foci. Figure 3A shows the resulting graphical representation of this quantification, presented as percentages relative to staining in uninjected embryos. *GATA4* staining is reduced to only 50% relative to uninjected and CMO injected embryos (p<0.001). In order to confirm specificity of loss-of-function, rescue experiments were performed using human-TSK, unaffected by the XMO that overlaps with the initiation site of *X-TSK*. Co-injection of 20 ng XMO with 1 ng *H-TSK* mRNA almost completely rescues X-TSK MO mediated inhibition of endoderm marker expression, *Sox17α* and *GATA4* (Figure 3A and Table 1) and partially restores numbers of *GATA4* positive foci (p<0.001, Figure 3A). Moreover, expression of 50 pg *Xnr2,* a known endoderm inducer [7] similarly restores expression of endoderm markers (p<0.001, Figure 3A). Interestingly, overexpression of H-TSK and Xnr2 increase levels of *GATA4* positive foci. Loss of endoderm upon X-TSK depletion is also clearly demonstrated in later stages where gut development is perturbed, with the gut becoming significantly thinner (21% reduction of measured gut width in comparison to uninjected embryos (p<0.001, Figure 3C). These observations in combination with specific expression of *X-TSK* in the endoderm indicate that X-TSK is a component of *Xenopus* endoderm formation.

**Table 1:** X-TSK Loss-of-Function

	Normal	Diminished	Expanded
***Sox17α***			
500 pg *β-Gal*	53 (100%)	0	0
20 ng CMO	44 (100%)	0	0
20 ng XMO	33 (59%)	23 (41%)	0
20 ng XMO+1ng *H-TSK*	26 (90%)	3 (10%)	0
20 ng XMO+50 ng *Xnr2*	29 (93.5%)	2 (6.5%)	0
***GATA4***			
500 pg *β-Gal*	28 (100%)	0	0
20 ng CMO	31 (100%)	0	0
20 ng XMO	16 (52%)	15 (48%)	0
20 ng XMO+1ng *H-TSK*	27 (90%)	3 (10%)	0
20 ng XMO+50 ng *Xnr2*	23 (82%)	5 (18%)	0
***Gsc***			
500 pg *β-Gal*	88 (98%)	1	1
20 ng CMO	59 (97%)	2	0
20 ng XMO	28 (48%)	30 (52%)	0
***MyoD***			
500 pg *β-Gal*	54 (100%)	0	0
20 ng CMO	43 (98%)	1	0
20 ng XMO	50 (77%)	0	15 (23%)

We continued by targeting XMO to a further *X-TSK* expressing region, the dorsal mesoderm, site of the organizer. Expression of the organizer gene *Gsc* is diminished upon X-TSK depletion in 52% of embryos, in agreement with TSK function as a BMP antagonist (Figure 3B). In order to analyze general mesoderm formation, we examined the effect of XMO on expression of the muscle marker *MyoD,* localized to ventrolateral mesoderm, and later in somites [42]. XMO was targeted to one side of the embryo, as marked by β-Gal staining (Figure 3B), thus acting as an internal control by which the proportion of uninjected vs. injected *MyoD* expression areas was measured using the Image J program. Figure 3B demonstrates a 30% expansion (p<0.001) in the area of *MyoD* expression, relative to the uninjected side, upon X-TSK depletion. We also analyzed expression of pan-mesoderm marker *Xbra,* in X-TSK depleted mesoderm. We found that *Xbra* expression is upregulated in XMO injected DMZ, as measured by RT-PCR (shown later). These effects in general mesoderm are subtle, perhaps due to redundancy with other SLRP family members, such as biglycan [43] or requirement of a positive factor to induce mesoderm. Nevertheless, this data does demonstrate a role for X-TSK in mesoderm formation and dorsal-ventral mesoderm patterning.

### X-TSK overexpression expands endoderm and dorsal mesoderm whilst inhibiting ventrolateral mesoderm formation

**Figure 4:**
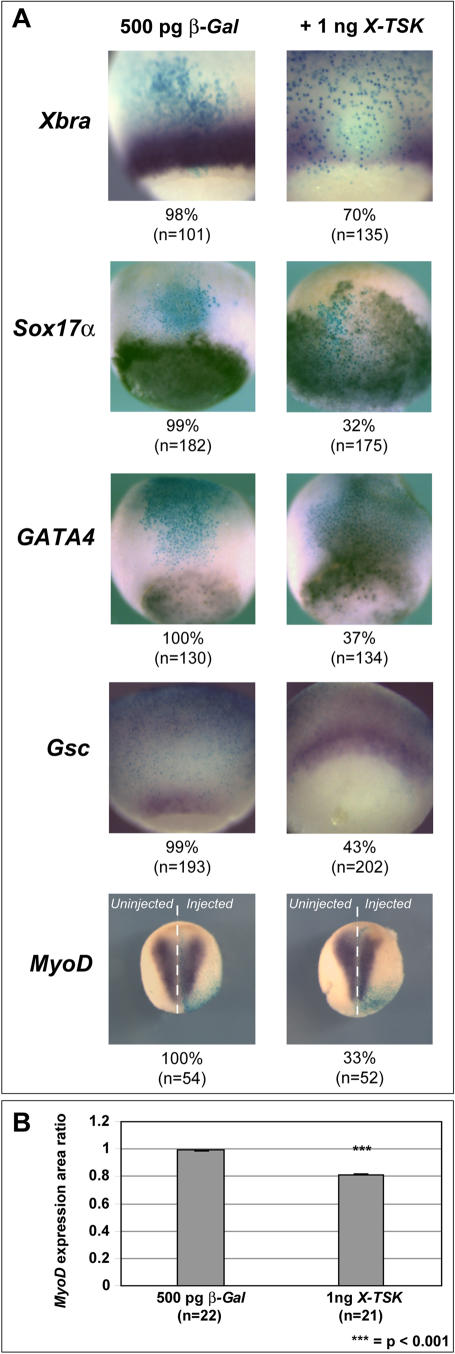
Gain of X-TSK function. (A) Whole mount in situ hybridization of germ layer markers in embryos injected with 500 pg *β-Gal* and 1 ng *X-TSK,* with percentage occurrence of demonstrated phenotype and ‘n’ numbers indicated below images. *Xbra* (pan-mesoderm) expression is inhibited and *Sox17α* and *GATA4* (endoderm) expression is expanded, stage 10.5, lateral orientation. *Gsc* (dorsal mesoderm) expression is expanded, stage 10.5, dorsal orientation. *MyoD* expression is inhibited on the injected side, as identified by blue *β-Gal* staining, stage 16, anterior top, posterior bottom. (B) Graphical representation of *MyoD* expression in (A). *MyoD* expression is reduced by 20% on the injected side (p = <0.001).

To support loss-of-function data, expression of germ layer markers was analyzed following overexpression of *X-TSK* in lateral marginal zone, an area of relatively low *X-TSK* expression, again with β-Galactosidase to identify the targeted area. Figure 4A shows that embryos injected with 1 ng *X-TSK* mRNA demonstrate substantial expansion of endoderm markers *Sox17α* (32% of embryos injected) and *GATA4* (37% of embryos injected), into the marginal zone. Although this phenotype is not penetrant, it is observed consistently. This data is also supported in loss-of-function rescues performed above where H-TSK expression increases numbers of *GATA4* foci in sectioned embryos by 31% (Figure 3A).

In contrast to this, pan-mesoderm *Xbra* expression was inhibited in 70% of embryos injected with 1 ng X-TSK (Figure 4A). Later in development, these embryos also demonstrate visibly reduced *MyoD* expression in 33% of embryos. More detailed analysis demonstrates an 18% reduction in area of *MyoD* expression on the injected side (p<0.001) (Figure 4B). This is also supported by the observation that gastrula-stage *MyoD* expression is inhibited by TSK overexpression (data not shown). In contrast to this, X-TSK overexpression in dorsal marginal zone (DMZ) clearly expands *Gsc* expression of the organizer in 43% of injected embryos (Figure 4A). Both loss- and gain-of-function analyses indicate that X-TSK acts as a component of endoderm and dorsal mesoderm formation, in addition to negative regulation of ventrolateral mesoderm. Specifically, X-TSK appears to function in *Xenopus* germ layer formation to induce endoderm and dorsal mesoderm, whilst inhibiting ventrolateral mesoderm formation. This hypothesis is supported by the observation that *X-TSK* is expressed within endoderm and dorsal mesoderm relative to much lower expression within ventrolateral mesoderm.

### X-TSK function in germ layer formation and patterning is not exclusively mediated by BMP inhibition

**Figure 5:**
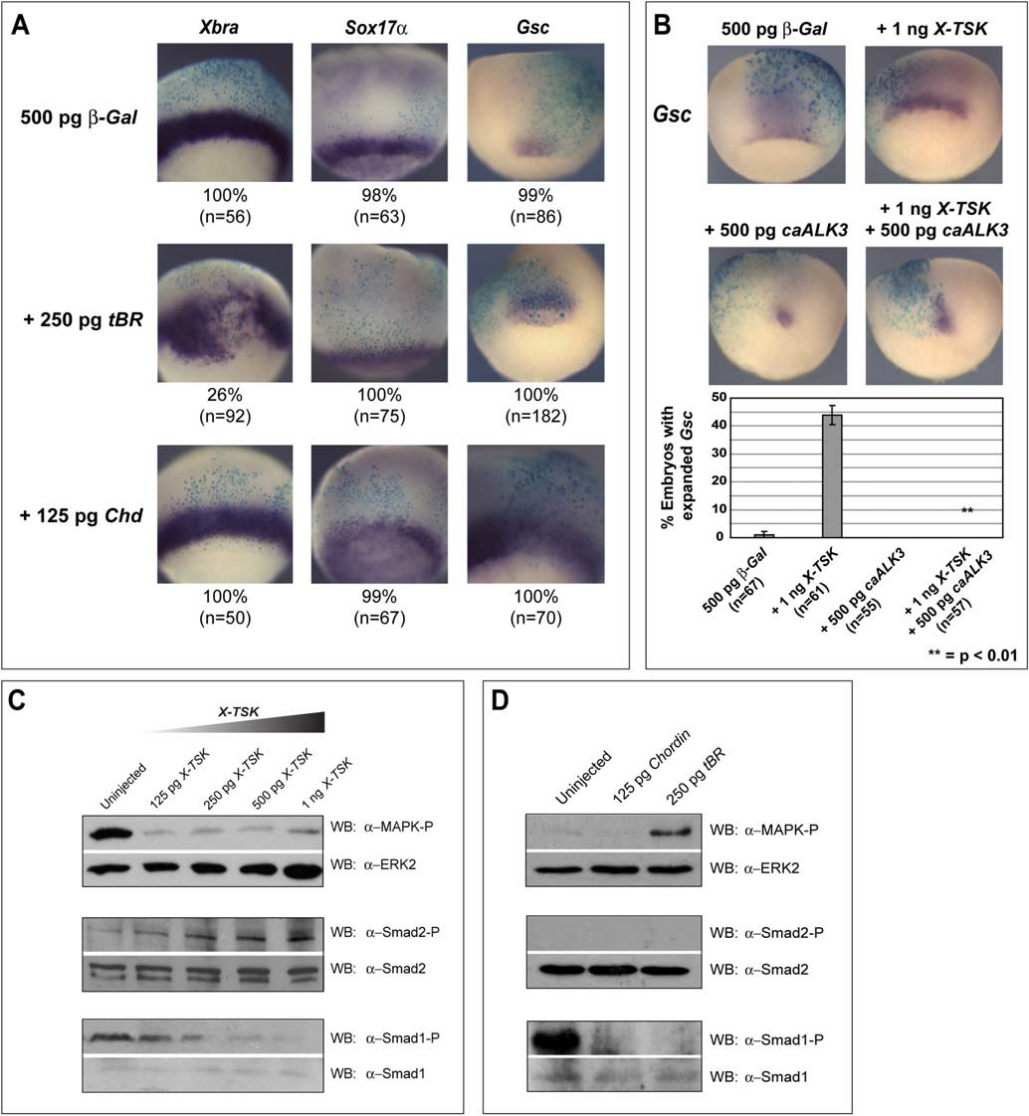
Mechanism of X-TSK function: signal analysis. (A) Whole mount in situ hybridization of germ layer markers in embryos injected with 500 pg *β-Gal* with 250 pg *truncated BMP receptor (tBR)* or 125 pg *Chordin (Chd),* with percentage occurance of demonstrated phenotype and ‘n’ numbers. *Xbra* and *Sox17α* phenotypes differ in comparison to *X-TSK* overexpression, whereas *Gsc* expression is commonly expanded. (B) Whole mount in situ hybridization of *Gsc* in embryos injected with 500 pg *β-Gal* with 1 ng *X-TSK*, 500 pg *caALK3* and *X-TSK* with *caALK3*, dorsal orientation. caALK3 blocks X-TSK mediated expansion of *Gsc* expression. (C) Western blotting of MAPK and Smad1 phosphorylation in animal caps and Smad2 phosphorylation in DMZ explants, with total MAPK, Smad2 and Smad1 controls in explants injected with *X-TSK* (125 pg-1 ng) (D) 125 pg *Chd* or 250 pg *tBR*. X-TSK inhibits MAPK and BMP phosphorylation in animal caps whilst activating Smad2 phosphorylation in DMZ. Chd and tBR similarly inhibit BMP phosphorylation, but contrast with X-TSK in MAPK and Smad2 phosphorylation status.

It was previously reported that TSK inhibits BMP in cooperation with chordin [40], thus raising the possibility that X-TSK functions in germ layer formation and patterning through this mechanism. To test this possibility, germ layer marker expression was analyzed in embryos where BMP signaling was compromised; embryos were injected with 250 pg truncated BMP receptor (*tBR)* [44] or 125 pg *chordin (Chd)* with β-Galactosidase as a targeting marker to the lateral or dorsal marginal zone (Figure 5A). Injection of *tBR* or *Chd* into ventral marginal zone (VMZ) at these doses induces secondary axes in 85–100% of embryos (data not shown) and expands expression of organizer marker *Gsc* in all embryos analyzed when targeted to the DMZ (Figure 5A). This is expected, as previously reported [44], [45] and demonstrates that BMP is indeed effectively inhibited at these doses. X-TSK similarly expands *Gsc* expression, and this combined with previous knowledge that BMP signals are inhibited by TSK strongly suggests BMP inhibition as the mechanism involved. To confirm this hypothesis, we co-overexpressed X-TSK in DMZ with the constitutively active BMP receptor caALK3 [46]. Indeed, X-TSK mediated expansion of *Gsc* expression is blocked by hyperactive BMP signaling from caALK3 (Figure 5B), confirming that X-TSK expands the organizer region through BMP inhibition. In contrast to this, expression of *tBR* within the lateral marginal zone expands *Xbra* expression, with no expansion of *Sox17α* (Figure 5A). *Chd* injection did not affect expression of *Xbra* or *Sox17α* (Figure 5A). In addition to this, X-TSK mediated inhibition of *Xbra* expression is not blocked by activation of BMP signaling by caALK3 (data not shown). These observations strongly indicate that X-TSK does not affect endoderm or ventrolateral mesoderm formation through inhibition of BMP signaling alone, suggesting that other pathways function within this context.

### X-TSK inhibits FGF-MAPK and BMP/Smad1 signaling whilst enhancing activin-like/Smad2 Signaling

Since inhibition of BMP signaling alone is unable to explain X-TSK function in germ layer formation, we considered the potential roles of other major signaling pathways involved in early embryogenesis. MAPK, Smad1 and Smad2, downstream of FGF, BMP and activin-like signaling respectively [47] were analysed by Western blotting in X-TSK overexpressing explants. At this point, we did not consider Notch signaling, the function of which is currently complex in germ layer formation, and will be discussed later. As shown in Figure 5C, X-TSK inhibits Smad1 phosphorylation in animal explants in a dose dependent manner, in accordance with its function as a BMP inhibitor. Interestingly, from a lower dose range, X-TSK strongly inhibits phosphorylation of MAPK in animal explants. For analysis of Smad2, X-TSK was overexpressed in DMZ as activin-like ligands are present in this region, in contrast to the animal cap. Figure 5C shows that X-TSK activates Smad2 phosphorylation in DMZ. To address the possibility that X-TSK regulates these pathways through inhibition of BMP signaling, *tBR* and *Chd* injected explants were also analyzed (Figure 5D), resulting in expected inhibition of Smad1 phosphorlylation in animal explants. tBR activates MAPK phosphorylation in animal explants as reported previously [48], whilst having no effect on Smad2 phosphorylation in DMZ, as shown in figure 5D. Chd does not inhibit MAPK phosphorylation in animal explants, which is also supported by the observation that *Xbra* expression, requiring intact FGF-MAPK signaling [22], is not affected upon Chd overexpression. These results demonstrate that the action of X-TSK upon MAPK and Smad2 is not mediated through BMP antagonism, and suggests potential mechanisms underlying X-TSK function in germ layer formation and patterning, which we have examined in further detail.

### X-TSK blocks ventrolateral mesoderm formation through binding and inhibition of FGF8b

**Figure 6:**
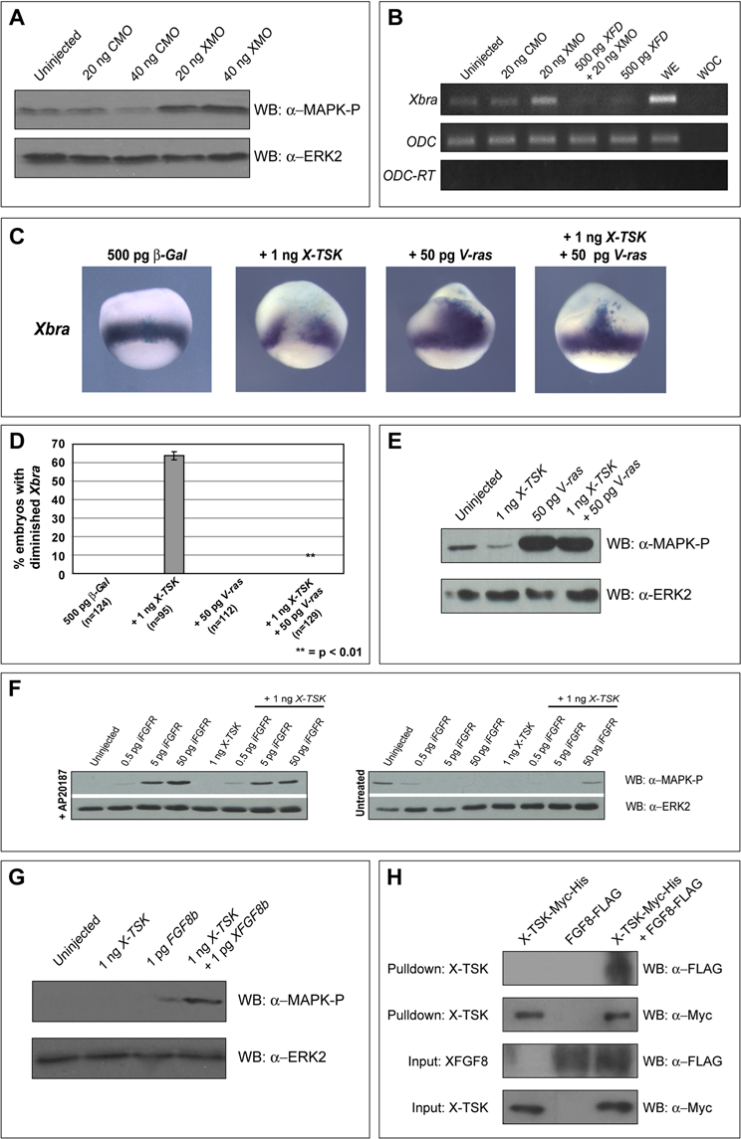
X-TSK inhibition and binding of FGF8b. (A) Western blotting of MAPK phosphorylation in animal caps injected with 20–40 ng CMO and 20–40 ng XMO. Depletion of X-TSK with XMO activates MAPK phosphorylation. (B) Semi-quantitative RT-PCR of *Xbra* expression in DMZ injected with 20 ng CMO, 20 ng XMO, 500 pg *XFD* and 500 pg *XFD* with 20 ng XMO. WE = Whole embryo, WOC = Water only control. Inhibition of FGF signals with XFD blocks *Xbra* expression activated upon depletion of X-TSK with XMO. (C) Whole mount in situ hybridization of *Xbra* in stage 10.5 embryos, lateral orientation. Embryos injected with 500 pg *β-Gal* with 1 ng *X-TSK*, 50 pg *V-ras* and *X-TSK* with *V-ras*. V-ras blocks X-TSK mediated inhibition of *Xbra* expression in 100% of embryos analyzed (p = <0.01), represented graphically in (D). (E) Western blotting of MAPK phosphorylation in animal caps injected with *X-TSK* and *V-ras*. V-ras blocks X-TSK mediated inhibition of MAPK phosphorylation. (F) Western blotting of MAPK phosphorylation in animal caps injected with *X-TSK* and *iFGFR,* in the presence or absence of chemical dimerisation agent, AP20187. Induced dimerisation blocks the activity of X-TSK to inhibit MAPK phosphorylation. (G) Western blotting of MAPK phosphorylation in animal caps injected with *X-TSK* and *FGF8b*. X-TSK inhibits MAPK phosphorylation activated by FGF8b. (H) Western blotting of nickel bead pulldown of FGF8b-FLAG in complex with X-TSK-Myc-His. Top panel: detection of FGF8b-FLAG in complex with X-TSK-Myc-His (third lane). Second panel: detection of X-TSK-Myc-His pulled down. Third and bottom panels: detection of FGF8b-FLAG and X-TSK-Myc-His input into the pulldown reaction.

A dominant negative FGF receptor (XFD) blocks mesoderm formation, demonstrating that intact FGF-MAPK signaling is required for mesoderm formation in *Xenopus* [22], [49]. However, Smad2 activation is known to enhance formation of mesoderm [50], ruling activin-like signaling out as a candidate in this context since X-TSK enhances Smad2 phosphorylation although inhibits general mesoderm formation. Therefore, we examined FGF-MAPK signaling as a mechanism for X-TSK mediated ventrolateral mesoderm inhibition. Figure 6A shows that MAPK phosphorylation is activated upon X-TSK depletion. Furthermore, upregulation of *Xbra* expression observed in X-TSK morphants is blocked by FGF inhibition with the dominant negative FGF receptor, XFD (Figure 6B). This demonstrates a role for endogenous X-TSK in inhibition of FGF signaling in the mesoderm.

We subsequently focused upon the point of X-TSK interaction within the FGF-MAPK pathway. We analysed the effect of V-ras, which functions as a constitutively active component of FGF-MAPK signaling, downstream of the FGF receptor [51]. Co-expression of V-ras with X-TSK and β-Galactosidase as a tracer completely blocks inhibition of *Xbra* expression by X-TSK and restores MAPK phosphorylation, as analyzed by Western blotting (Figure 6C–E), suggesting that X-TSK inhibits ventrolateral mesoderm formation through inhibition of FGF-MAPK signaling, upstream of Ras. Moreover, Figure 6G shows that MAPK activation by the potent mesoderm inducer FGF8b [52] is strongly inhibited by X-TSK. In support of this receptor-ligand level interaction, an inducible FGF receptor (iFGFR), which works as a constitutively active FGF receptor in the presence of dimerisation activator, AP20187 [53], rescues X-TSK mediated MAPK inhibition (Figure 6F).

These observations suggest that X-TSK inhibits FGF-MAPK activity at the extracellular level. FGF8b appeared as a good candidate in the mechanism of X-TSK mediated FGF-MAPK inhibition, hence ventrolateral mesoderm inhibition, thus we analyzed interaction between these proteins in a pulldown assay. Indeed FGF8b-FLAG is pulled down in complex with X-TSK-Myc-His in a reaction with nickel beads (Figure 6H). This interaction is specific, as FGF8b-FLAG in isolation is unable to bind nickel beads. In addition to this, the interaction with X-TSK is through FGF8b itself and not the FLAG epitope, as we have previously shown that X-TSK does not interact with activin-FLAG, or noggin-FLAG in the equivalent pulldown assay [40]. In combination, this evidence demonstrates that X-TSK inhibits ventrolateral mesoderm formation though binding and inhibition of FGF8b. The next part of the study focused upon the mechanism underlying endoderm induction by X-TSK.

### X-TSK induces endoderm through binding and enhancing activity of Xnr2

Data so far has shown that X-TSK functions as an endoderm inducer in *Xenopus*. Nodal, a member of the TGF-β superfamily, is known to induce endoderm at high concentrations through activation of Smad2 [54]. A total of six nodal related genes have been identified in *Xenopus* (*Xnr1-Xnr6*) [7], [8], [55], [56]. With the exception of Xnr3, they all demonstrate conserved functions upon overexpression, although their spatial and temporal expression varies. Of these Xnr family members, *Xnr2* expression overlaps most closely with *X-TSK* expression [7]. As shown in Figure 5C, X-TSK activates Smad2 phosphorylation. These observations suggested Xnr2-Smad2 signaling as a candidate mechanism for X-TSK mediated endoderm induction. Therefore, we analyzed the role of Xnr2 in the context of X-TSK function.

**Figure 7:**
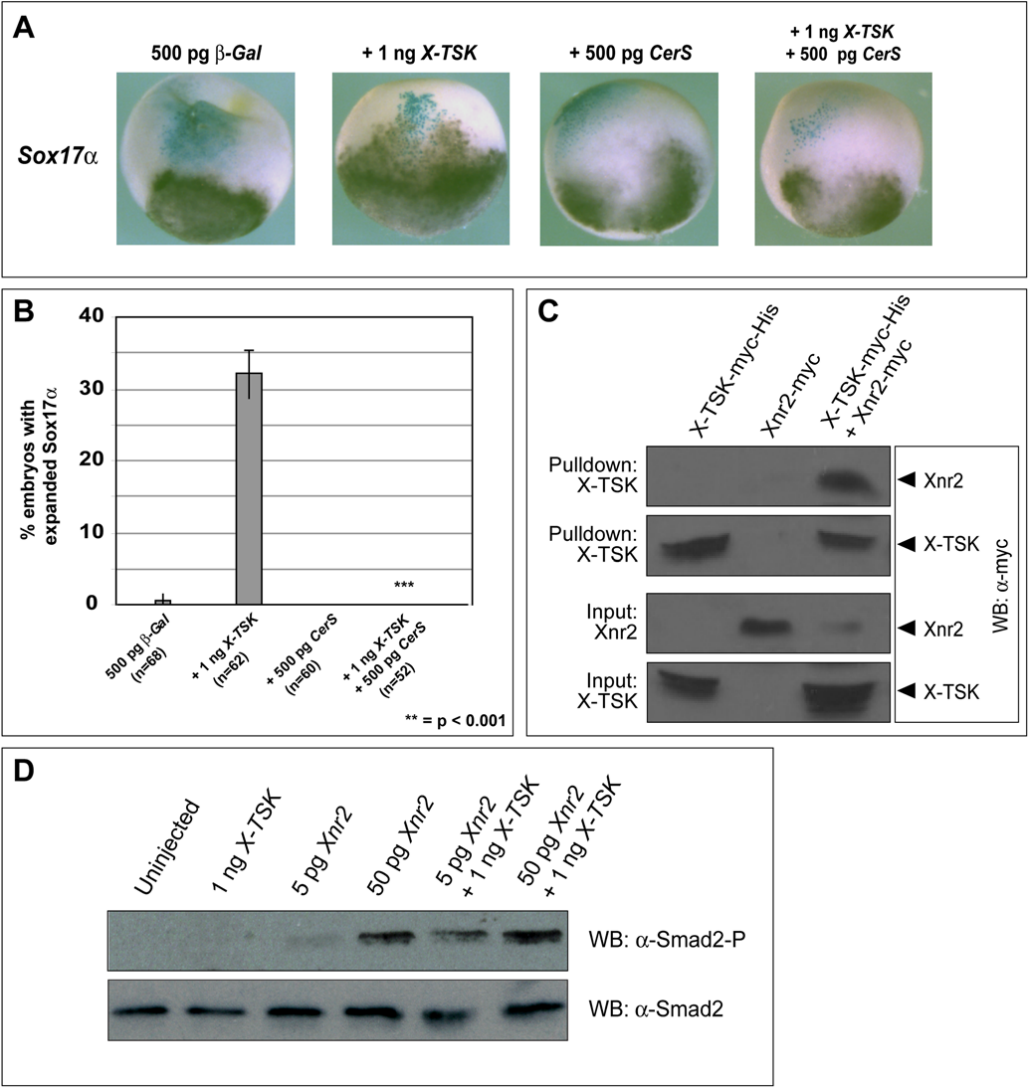
X-TSK requires intact Xnr signaling for endoderm induction; X-TSK binds to and enhances Xnr2 Signaling. (A) Whole mount in situ hybridization of *Sox17α* in embryos injected with 500 pg *β-Gal* with 1 ng *X-TSK,* 500 pg *CerS* and 500 pg *CerS* with 1 ng *X-TSK,* lateral orientation. (B) Introduction of CerS blocks X-TSK expansion of *Sox17α* in 100% of embryos analyzed (p = <0.001). (C) Western blotting of nickel bead pulldown of Xnr2-Myc in complex with X-TSK-Myc-His. Top panel: detection of Xnr2-Myc in complex with X-TSK-Myc-His (third lane). Second panel: detection of X-TSK-Myc-His pulled down. Third and bottom panels: detection of Xnr2-Myc and X-TSK-Myc-His input into the pulldown reaction. (D) Western blotting of Smad2 phosphorylation in animal caps injected with 1 ng *X-TSK*, 5 pg and 50 pg *Xnr2*. X-TSK enhances Smad2 phosphorylation, particularly evident with 5 pg Xnr2.

We questioned whether X-TSK requires intact Xnr signaling for endoderm induction. Using a truncated Cerberus mutant (CerS) that specifically inhibits Xnr signaling in *Xenopus* [8] we studied the effect of Xnr inhibition upon X-TSK mediated induction of endoderm by in situ hybridization. Introduction of CerS inhibits expression of endoderm marker *Sox17α* in all embryos analyzed. In the presence of CerS, X-TSK mediated endoderm formation is completely blocked (Figure 7A and 7B). Furthermore, 50 ng *Xnr2,* co-injected with XMO, visibly restores expression of *Sox17α* and *GATA4,* and GATA4 positive foci diminished upon loss of X-TSK function (Figure 3A). These data strongly indicate that intact Xnr signaling is required for X-TSK mediated endoderm induction.

**Figure 8:**
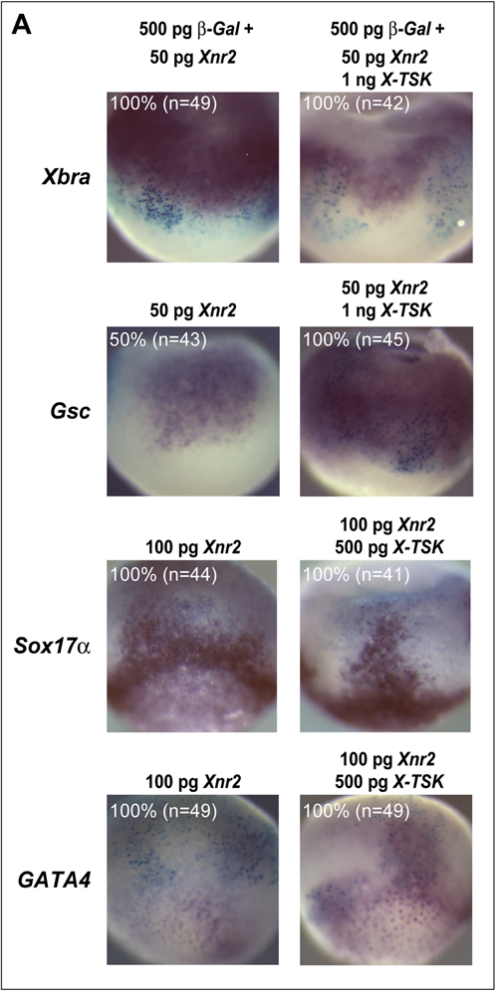
X-TSK changes local response to Xnr2. (A) Whole mount in situ hybridization of *Xbra, Gsc* (dorsal orientation) *Sox17α* and *GATA4* in embryos injected with 500 pg *β-Gal* with 50 pg *Xnr2,* and 50 pg *Xnr2* with 1ng *X-TSK* and 500 pg *X-TSK,* lateral orientation. *Xbra* expression is not detected in *Xnr2-X-TSK* expressing cells, as identified by *β-Gal* staining. *Xnr2* mediated expansion of *Gsc* expression is enhanced by X-TSK. Expression of endoderm markers *Sox17α* and *GATA4* expanded by *Xnr2* is enhanced by X-TSK, suggesting that X-TSK changes local cellular response to Xnr2.

Our previous observations that TSK binds to TGF-β superfamily members [35], [40] suggests that X-TSK may regulate Xnr activity through binding to Xnr proteins. As shown in Figure 7C, mature Xnr2-Myc is pulled down in complex with X-TSK-Myc-His in a reaction with nickel beads. This interaction is specific, as Xnr2-Myc in isolation is unable to bind the nickel beads. Moreover, the interaction with X-TSK is through Xnr2 itself and not the Myc epitope, as we have previously shown that X-TSK does not interact with ADMP-Myc, or Follistatin-Myc in equivalent pulldown assays [40]. As shown in Figure 7D, X-TSK enhances Xnr2 mediated Smad2 phosphorylation in animal cap explants; this is further supported by co-overexpression of *X-TSK* and *Xnr2* in whole embryos, where dorsal mesoderm and endoderm formation is enhanced, as marked by *Gsc, Sox17α* and *GATA4* respectively (Figure 8A). This data, along with rescue of X-TSK loss-of-function by Xnr2, demonstrates that X-TSK binds to and enhances *Xnr2,* where intact Xnr signaling is required for the function of X-TSK in endoderm induction.

Overexpression of Xnr2 in *Xenopus* embryos strongly activates expression of pan-mesoderm marker *Xbra*, in addition to expansion of dorsal marker *Gsc*, and endoderm markers *Sox17α* and *GATA4* (Figure 8A). However, in these cases, Xnr2 induces both endoderm and mesoderm markers without forming a clear border between them. Also the induction of *Xbra* in particular is detected far beyond Xnr2 expressing cells, as identified by β-Galactosidase staining. Very interestingly, co-overexpression of Xnr2 and X-TSK induces *Xbra* expression only outside the targeted region. Expression of *Xbra* within injected cells is almost completely inhibited whilst endoderm marker expression is enhanced. These observations indicate that X-TSK exerts its effects at short-range, while Xnr2 functions over a long range [10]. This idea of X-TSK acting at short range is also supported by our previous observations; TSK is secreted from cultured cells and has no membrane spanning domain or GPI anchoring signal [33], and X-TSK fused with CD2, a membrane linker, showed an identical activity to wild type X-TSK in neural crest development [34].

### Combinatorial regulation of BMP, FGF and Xnr signaling by X-TSK potentiates endoderm formation

**Figure 9:**
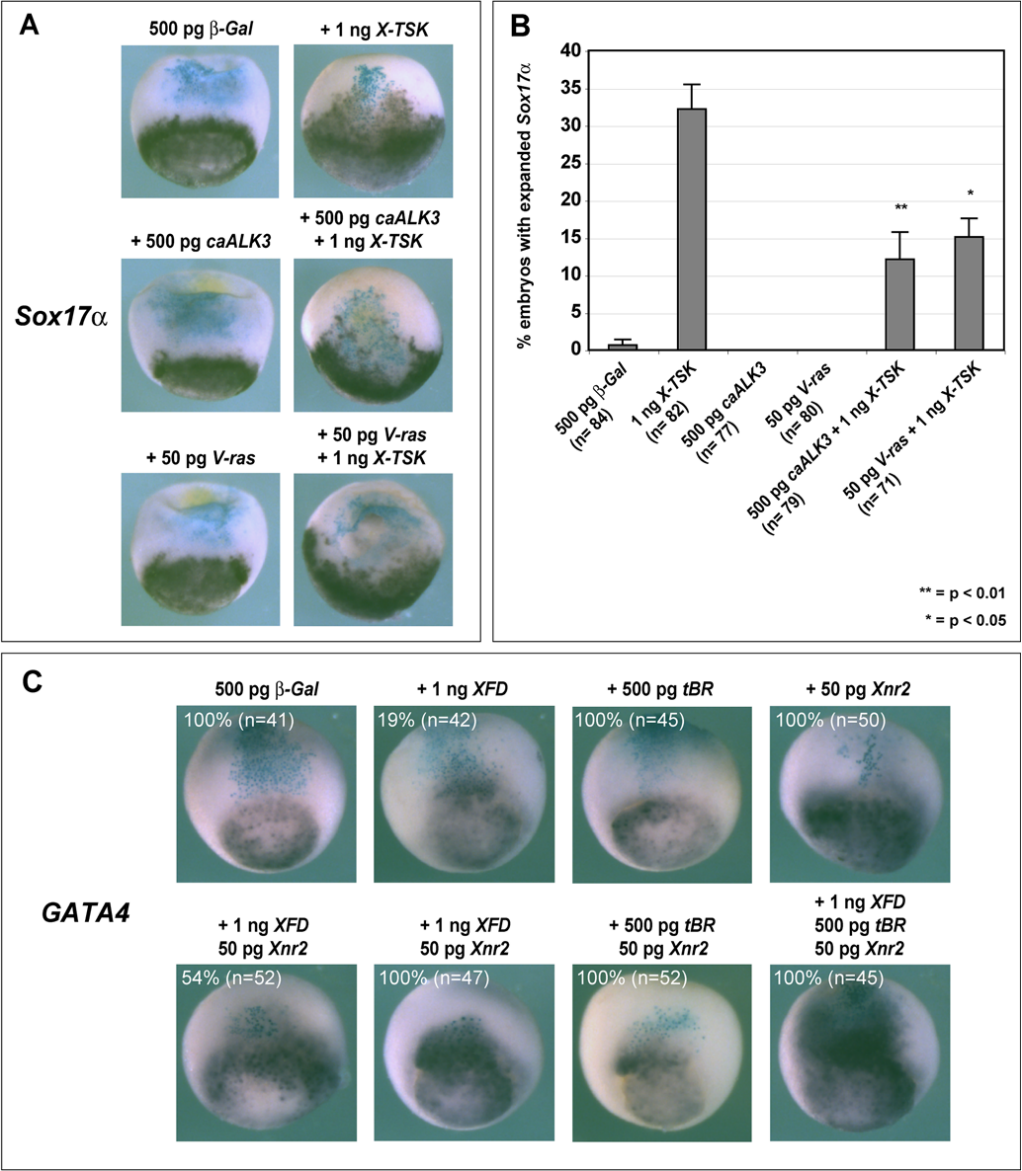
BMP and FGF signal activation blocks X-TSK mediated endoderm induction: triple signal regulation. (A) Whole mount in situ hybridization of *Sox17α* in embryos injected with 500 pg *β-Gal* with 1 ng *X-TSK,* 500 pg *caALK3,* 50 pg *V-ras* and 500 pg *caALK3,* 50 pg *V-ras* with 1 ng *X-TSK,* lateral orientation. (B) Graphic representation of quantity of embryos demonstrating expanded *Sox17α* expression. Introduction of caALK3 and V-ras partially blocks X-TSK expansion of *Sox17α* (p = 0.01 and 0.05 respectively). (C) Whole mount in situ hybridization of *GATA4* in embryos injected with 500 pg *β-Gal* with combinations of 1 ng *XFD,* 500 pg *tBR,* and 50 pg *Xnr2,* lateral orientation. Demonstrated phenotype frequencies with n-numbers in white text. A triple combination of 1 ng *XFD,* 500 pg *tBR,* and 50 pg *Xnr2* produces the strongest expansion of *GATA4* expression.

We have shown that X-TSK binds to and modulates the activity of BMP, FGF8b and Xnr2 in a concentration dependent manner. Although we have demonstrated that X-TSK regulation of Xnr2 is required for endoderm formation, a previous study with chordin and dominant negative FGFR (XFD) has shown that BMP and FGF inhibition also participate in endoderm formation [26]. Thus, we examined the effect of BMP and FGF-MAPK activation upon X-TSK mediated endoderm formation (Figure 9A and 9B). FGF-MAPK activation by V-ras partially blocks X-TSK mediated endoderm formation (reduced to 15% from 32%, p<0.05). In addition to this, BMP activation by caALK3 also partially blocks X-TSK mediated endoderm formation (reduced to 12% from 32%, p<0.01). This demonstrates that X-TSK activates endoderm formation through extracellular coordination of three pathways: Xnr2, FGF-MAPK and BMP.

To confirm the importance of multiple signal integration, we analyzed the combined effects of BMP and FGF signal inhibition with Xnr2 signal activation upon endoderm induction. Expression of truncated BMP receptor (*tBR*) in lateral marginal zone did not expand expression of endoderm marker *GATA4* (Figure 9C), whereas expression of dominant negative FGF receptor (*XFD*) or *Xnr2* produces only a light, diffuse expansion of *GATA4* expression. However, combinations of *XFD-tBR, XFD-Xnr2* and *tBR-Xnr2* produced a stronger activation of *GATA4* expression. Interestingly, combination of *tBR, XFD,* and *Xnr2* produced a much stronger activation of *GATA4* expression, indicating that inhibition of both FGF and BMP signaling pathways in combination with Xnr activation is important for endoderm formation, supporting the observed multiple signal regulation by X-TSK.

### Zygotic expression of TSK is regulated by FGF-MAPK signaling

**Figure 10:**
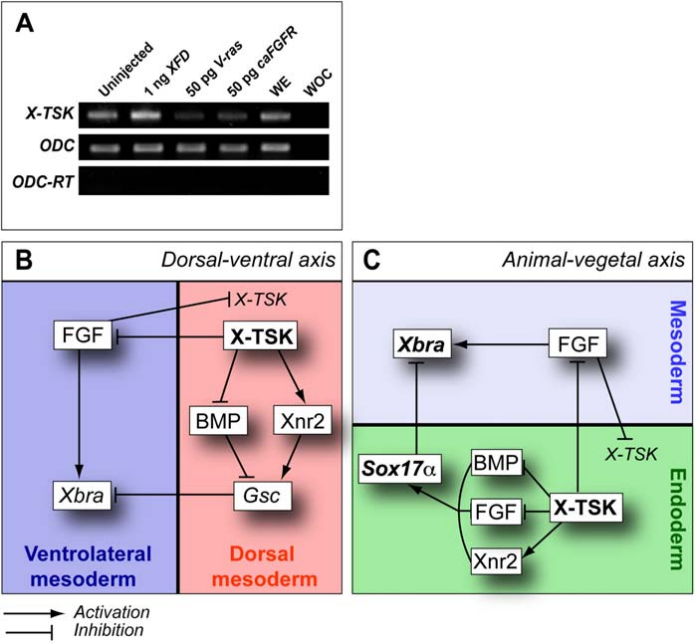
Transcriptional regulation of *X-TSK* and model of X-TSK function in germ layer formation and patterning. (A) Semi-quantitative RT-PCR of *X-TSK* expression in animal caps injected with 1 ng *XFD,* 50 pg *V-ras* or 50 pg *caFGFR.* WE = Whole embryo, WOC = Water only control. Inhibition of FGF signals with XFD enhances *TSK* expression, whereas activation of FGF signals with V-ras or caFGFR reduces *TSK* expression levels. (B) Model of TSK function in *Xenopus* germ layer formation and patterning: dorsal-ventral mesoderm patterning. X-TSK in dorsal mesoderm (red) inhibits BMP signaling to promote dorsal mesoderm formation, as marked by *Gsc* expression. This is possibly also enhanced through activation of Xnr2 signals by TSK. MAPK activation inhibits *X-TSK* expression in ventrolateral mesoderm, where X-TSK inhibits expression of ventrolateral mesoderm markers such as *Xbra*, through inhibition of FGF signaling. This network of signaling may contribute to clear patterning of the mesoderm. (C) Model of TSK function in endoderm formation. X-TSK coordinates inhibition of FGF and BMP signals with activation of Xnr2 signaling to induce endoderm formation (green), as marked by *Sox17α.* Again, X-TSK inhibits expression of ventrolateral mesoderm (blue) markers such as *Xbra*, through inhibition of FGF signaling and may contribute to the distinction between endoderm and mesoderm specific gene expression.

Our data indicates that germ layer formation and patterning is influenced by expression of *X-TSK*, suggesting the importance of *TSK* transcriptional regulation. As shown in Figure 2, *X-TSK* possesses a dynamic and unique expression pattern during early embryogenesis. Zygotic transcription of *X-TSK* is initiated in the dorsal region, followed by expression in the endoderm, with exclusion from ventrolateral mesoderm. This region of exclusion corresponds to an area of high FGF activity [57]. Therefore, the effect of FGF-MAPK activity upon zygotic X-TSK expression was studied by semi-quantitative RT-PCR. As shown in Figure 10A, activation of FGF-MAPK signaling with V-ras or constitutively active FGF receptor (caFGFR) inhibits X-TSK expression, whilst its inhibition with XFD activates *X-TSK* expression. These findings suggest that *X-TSK* expression is shaped by FGF signaling, where a feedback loop may be formed by which X-TSK inhibits FGF signaling at the extracellular level.

## Discussion

In this paper, we describe three major functions of X-TSK: activation of endoderm formation, inhibition of ventrolateral mesoderm formation, and expansion of dorsal mesoderm. These functions are mediated by multiple signal integration; inhibition of FGF-MAPK and BMP signaling, with enhancement of Xnr2 signaling by X-TSK, regulated in the extracellular space through protein-protein interactions.

### TSK in mesoderm formation and patterning

We have shown previously that in chick, TSK functions as an organizer inducer [33]. In *Xenopus*, *X-TSK* is expressed in the dorsal blastopore lip where Spemann's organizer is located. Functional analysis presented in this current study shows that X-TSK has activity to expand the region expressing the organizer gene *Gsc,* mediated through BMP inhibition. In addition to this, we have previously shown that X-TSK directly induces neural tissue in animal caps and expands the neural region in whole embryos [33], [34]. These observations indicate that X-TSK is working as a component of the organizer. However, X-TSK has two additional functions that differentiate it from other organizer molecules: induction of endoderm and inhibition of ventrolateral mesoderm. *X-TSK* is excluded from ventrolateral mesoderm, mediated by FGF signaling. Overexpression of X-TSK within the marginal zone inhibits expression of ventrolateral mesoderm markers, mediated through inhibition of FGF8b in the extracellular space by X-TSK.

It is known that pan-mesoderm markers such as *Xbra* require intact FGF signaling for their expression [22], whilst expression of dorsal organizer markers such as *Gsc* is not dependent on FGF signal status [23]. Therefore, it is possible to activate organizer formation whilst FGF signals are inhibited, although in vivo signal analysis in *Xenopus* embryogenesis showed that MAPK is activated in the organizer [57]. Since *X-TSK* inhibits FGF-MAPK and is expressed within the organizer, it is likely there may be a mechanism in place to interfere with or reduce X-TSK inhibition of FGF-MAPK in this region.

### The role of X-TSK in endoderm induction

In this study, we have demonstrated that X-TSK is a component of endoderm induction in *Xenopus*. Zygotic expression of *X-TSK* is activated in endoderm, overlapping spatially and temporally with *Xnr2* expression [7]. Our functional analysis has shown that X-TSK physically interacts with Xnr2, and enhances Smad2 activity downstream of Xnr2. In addition to this, X-TSK has two more activities: binding and inhibition of FGF8b, in addition to previously reported BMP binding and inhibition. It is known that strong activation of nodal signaling plays a major role in endoderm induction in all vertebrates [58]. In combination with this, FGF and BMP inhibition also function to positively contribute to endoderm induction [26]. Our experiments using Xnr2, tBR, and XFD clearly show that coordination of the three pathways has much stronger endoderm inductive activity relative to manipulation of individual or dual signaling pathway components. We have demonstrated that X-TSK is a key coordinator of these multiple pathways outside the cell through regulation of an extracellular signaling network. In terms of the intracellular pathways involved in endoderm induction downstream of X-TSK activity; it is known that transcription factors such as Sox17, Mixer, Mix1, and Bix2/milk have dual activities that inhibit mesoderm formation and activate endoderm formation [15], [20], [59]. It is likely that such transcription factors are activated downstream of signaling coordinated by X-TSK, further refining germ layer specific gene expression.

It is important to note that X-TSK overexpression in animal caps produces only a weak induction of *Sox17α* as measured by in situ hybridization and RT-PCR (data not shown). This induction may be weak as FGF signals are activated upon dissection, in addition to the important point that activin-like ligands are not present in the animal region. Moreover, the demonstrated induction of endoderm markers upon X-TSK overexpression is consistent, although not penetrant. This may be due to the fact that many natural mechanisms are in place to prevent induction of endoderm within the marginal zone.

There are significant differences in mesoderm and endoderm formation between different organisms, although recently, conserved molecular mechanisms have been elucidated [60]. This raises the possibility that TSK may have a conserved function between species. In support of this, we have demonstrated similar roles for TSK in organizer formation in chick and *Xenopus.* In chick, *TSK* is expressed in the posterior marginal zone and Koller's sickle, followed by expression in the extending primitive streak and Hensen's node; whilst in zebrafish, *TSK* is expressed in the blastoderm margin [33], structures composed of both mesoderm and endoderm precursors. Furthermore, in the case of chick, *nodal* is expressed in Koller's sickle [60], suggesting that TSK may work with nodal to regulate specification of mesoderm and endoderm. More evidence of conservation of mechanism across species can be found in zebrafish. Here we have shown that coordinated modulation of BMP, FGF and Xnr pathways is important in *Xenopus* endoderm formaton; this is also true for the zebrafish where combined BMP, FGF and nodal signaling have been shown to regulate endoderm formation and segregation of endoderm and mesoderm precursors [61]. Future studies into the potential conservation of TSK function between species may prove to be interesting and may also provide more information on conservation of signaling involved.

### Integration of multiple signaling pathways

Temporal and spatial regulation of multiple signaling pathways is essential for tightly controlled regulation of development. Secreted soluble growth factors and their inhibitors have fundamental roles in signal regulation; however, studies about coordination of these pathways are largely restricted to intracellular cross talk [62], yet several extracellular regulators such as follistatin and cerberus are known to interact with multiple signaling pathways. Our analyses have demonstrated that X-TSK binds to and regulates FGF8b, BMP and Xnr2 at the extracellular level, in a concentration dependent manner to function in germ layer formation and patterning. Our previous work suggests that TSK function is not restricted to endoderm and mesoderm; in ectoderm, X-TSK regulates BMP activity, which contributes to a decision between epithelial and neural tissues. In addition to this, X-TSK regulates Delta-Notch signaling during neural crest formation [34]. Currently, the function of Notch in mesoderm formation is complex, where some evidence suggests that activation of Notch contributes to mesoderm inhibition whilst endoderm is induced. Conversely, it has been found that activation of Notch signaling delays loss of mesodermal competence [63], [64]. Although we cannot rule out participation of the Notch pathway, which may prove to be interesting in future studies, we did not study Notch signaling in the context of TSK mediated germ layer formation and patterning due to these complexities. The potential participation of Vg1 must also be considered in future studies, as TSK activates Vg1 in chick development [35]. In *Xenopus,* it had been thought that Vg1 was not processed until a second allele was identified [65], although no effect on endoderm has been reported in loss-of-function studies. Therefore, Xnr2 remained a more attractive candidate in TSK functional mechanism in the endoderm and dorsal mesoderm. Although we have shown here that intact Xnr2 signaling is indeed required for TSK function, future work with Vg1 will be interesting. Even so, we have clearly demonstrated the involvement of FGF, BMP and Xnr pathways. Our analysis shows the importance of extracellular cross talk of these pathways and suggests that extracellular coordination of multiple signaling pathways may have important roles in cell signaling. This is supported by the potential involvement of additional signaling pathways, including Notch and Vg1.

### Signal coordination in germ layer formation

Morphogens such as activin-like proteins have been shown create distinct fates depending on their concentrations [66]. It has been reported that cellular response to activin depends on the absolute number of receptors occupied by activin [67], indicating that regulation of morphogen diffusion is critical to create an appropriate concentration gradient. This importance of diffusion control has been demonstrated recently; *Drosophila* mutants *toutvelu* and *dally*, which have defects in the synthesis of extracellular heparan sulfate proteoglycans, demonstate defective Wingless morphogen diffusion [68]. Members of the SLRP family are proteoglycans [69] and thus may regulate morphogen gradients in a similar way.

Here we propose a model for TSK function in *Xenopus* germ layer formation and patterning. With the combination of X-TSK and Xnr2, there is no overlap between endoderm markers and pan-mesoderm marker *Xbra*. Thus it is tempting to speculate that TSK may contribute to segregation between endoderm and mesoderm specific gene expression by a combination of four factors. Firstly, Xnr2 and X-TSK have distinct functional ranges with Xnr2 working as a long-range morphogen [70], whilst X-TSK works at short-range [34]. Secondly, Xnr2 and X-TSK regulate distinct sets of signaling pathways. Thirdly, X-TSK functions with several factors to synergistically potentiate or inhibit the activities of these proteins. This interaction may change the effective concentration or diffusion of proteins. Thus, in the case of co-overexpression, X-TSK in proximity to cells expressing the two proteins creates a competent area; here X-TSK makes a complex with Xnr2 and potentiates signaling whilst inhibiting FGF-MAPK and BMP signaling. This coordinated regulation may produce clear regions of gene expression.

We need to consider a fourth important factor: spatial and temporal transcriptional regulation. Zygotic *X-TSK* is expressed in endoderm, dorsal mesoderm, and ectoderm with exclusion from ventrolateral mesoderm. Zygotic *X-TSK* expression in ventrolateral mesoderm is inhibited by FGF-MAPK signaling. Within the endoderm, a regulatory loop may be created in which TSK inhibits FGF-MAPK, which in turn promotes TSK expression. Based on our observations, we propose a model for X-TSK mediated embryonic patterning (Figure 10B). In the absence of X-TSK, Xnr proteins create a pattern of Smad2 activation with a vegetal-animal gradient. Activated *X-TSK* expression in the endoderm creates a competent area, in which the activity of Xnr proteins is enhanced, possibly by an increase in local effective concentration. X-TSK provides ideal coordination for endoderm formation: Xnr2 activation, FGF-MAPK inhibition, and BMP inhibition. Conversely, MAPK activation inhibits expression of *X-TSK* in ventrolateral mesoderm, possibly permitting mesoderm-specific gene expression in this area. Moreover, *X-TSK* expression in dorsal mesoderm contributes to organizer formation and function, mainly through BMP inhibition by synergistic ternary complex formation among X-TSK, BMP, and chordin [40] and possibly through Xnr activation. In conclusion, through regulation of these multiple factors, TSK coordinates formation of the endoderm and patterning of mesoderm during early *Xenopus* embryogenesis.

### Future perspectives

It is now becoming clear that future work will focus upon the dissection of an ‘extracellular network’ of signal regulation, unraveling its importance in cell signaling as a whole. We have demonstrated the importance of extracellular coordination of FGF, Xnr2 and BMP signals in germ layer formation and patterning. Future work will involve the potential role of Notch and other activin-like signaling, which may go to demonstrate the nature of extracellular networks in greater detail. It will also be important to unravel the function of TSK in germ layer formation and patterning in other species such as chick and zebrafish, in order to demonstrate conservation and the importance of extracellular coordination of multiple pathways.

## Materials and Methods

### Embryology and in situ hybridization

*Xenopus laevis* embryos were staged according to Nieuwkoop and Faber [71]. For animal cap assays, mRNA was injected into the animal pole of 2-cell stage embryos. Animal caps were dissected from stage 8-9 embryos in 1× MBS and cultured in 0.7× MBS. For DMZ assays, mRNA was injected into the DMZ of 8-cell stage embryos. DMZ was dissected from stage 10 embryos in 1× MBS and cultured in 0.7× MBS. Whole mount in situ hybridization was performed as described [72]. In situ hybridization of sectioned embryos was performed as described [36]. The following probes were used; *X-TSK, Xbra* [39], *MyoD* [42], *Gsc* [38], *Sox17α* [37], *GATA4* [41]. Gut width and areas of *MyoD* expression were measured using Image J software (NIH). T Tests were performed to evaluate the statistical significance of results.

### Semi-quantitative RT-PCR

Embryos, animal caps or DMZ explants at the indicated stages were snap-frozen, followed by RNA isolation with RNeasy kit (Qiagen). cDNAs were generated according to the manufacturers protocol (Taqman RT Reagents, Applied Biosystems). Primers used for the PCR reaction were described previously: *ODC* [18], *Xbra* [18], *X-TSK* [34]. Quantitative ranges were determined before final analysis. All reactions were normalized against *ODC* gene product.

### Microinjection of mRNA or morpholino oligonucleotides

Capped mRNAs were synthesized from linearized plasmid templates with mMessage Machine (Ambion). Embryos were injected with 1-1000 pg mRNA per embryo at the indicated stages in 0.2× MBS with 4% Ficoll. The following mRNAs were synthesized: *X-TSK* (*pCS2+X-TSK*) and *X-TSK-myc-His* [33], [34], *H-TSK* (*pCS2+H-TSK,* accession number AF191019), *truncated BMP receptor* (*tBR*) [44], *Chordin* [45], *Xnr2* and *Xnr2-myc* [7], *V-ras* [51], *CerS* [8], *iFGFR* [53], *FGF8b* [52], *caALK3* [46]. A morpholino antisense oligonucleotide (MO) against *X-TSK* was used for loss-of-function experiments and has previously been shown to specifically deplete X-TSK [34].

### Pulldown assays

X-TSK-myc-His, FGF8b-FLAG, Xnr2-Myc and FRL1-FLAG were expressed in *Xenopus* embryos and COS7. Stage 10.5 embryos were lysed in IP buffer [73]. After centrifugation, the soluble fraction was used for pulldown assay as previously described [33]. Myc-tagged and FLAG-tagged proteins were detected after blotting using an anti-myc antibody 9E10 or anti-FLAG antibody M2 (Sigma), anti-mouse-HRP (Amersham).

### Cell signaling assays

*Xenopus* embryos were microinjected at the two-cell stage and incubated until stage 8. Animal caps were dissected as above and cultured until stage 10. DMZ was explanted at stage 10. Phospho-Smad1, phospho-Smad2 and phospho-MAPK analysis was performed as previous [74]. The following antibodies were used; α-Activated clone MAPK-YT (Sigma), α-ERK (BD Biosciences), α-Phospho-Smad2 (Cell Signaling Technology), α-Smad2 (BD Biosciences), α-Phospho-Smad1 (Cell Signaling Technology), and α-Smad1 (Santa Cruz).
